# The Optimal Cut-off Score of the Nijmegen Questionnaire for Diagnosing Hyperventilation Syndrome Using a Bayesian Model in the Absence of a Gold Standard

**DOI:** 10.31661/gmj.v9i0.1738

**Published:** 2020-06-24

**Authors:** Mehdi Azizmohammad Looha, Fatemeh Masaebi, Mohsen Abedi, Navid Mohseni, Atefeh Fakharian

**Affiliations:** ^1^Department of Biostatistics, Faculty of Paramedical Sciences, Shahid Beheshti University of Medical Sciences, Tehran, Iran; ^2^Physiotherapy Research Centre, School of Rehabilitation Sciences, Shahid Beheshti University of Medical Sciences, Tehran, Iran; ^3^Chronic Respiratory Diseases Research Center, Shahid Beheshti University of Medical Sciences, Tehran, Iran

**Keywords:** Hyperventilation, Questionnaire, Sensitivity, Specificity

## Abstract

**Background:**

The Nijmegen questionnaire is one of the most common tools for diagnosing hyperventilation syndrome (HVS). However, there is no precise cut-off score for differentiating patients with HVS from those without HVS. This study was conducted to evaluate the accuracy of Nijmegen questionnaire for detecting patients with HVS and to provide the best cut-off score for differentiating patients with HVS from normal individuals using a Bayesian model in the absence of a gold standard.

**Materials and Methods:**

A total of 490 students from a rehabilitation center in Tehran, Iran, were asked to participate in this case study of HVS from January to August 2018.

**Results:**

A total of 215 students (40% male and 60% female) completed the Nijmegen questionnaire. The area under the receiver operating characteristic curve (AUC) was 0.93 (male: 0.95; female: 94) for all of the cut-off scores. The optimal cut-off score of ≥20 could predict HVS with sensitivity of 0.91 (male: 0.99; female: 91) and specificity of 0.92 (male: 96; female: 89).

**Conclusion:**

Accurate differentiation of HVS patients from individuals without HVS can be accomplished by estimating the cut-off score of Nijmegen questionnaire based on a non-parametric Bayesian model.

## Introduction

Dysfunctional breathing (DB), as a multidimensional disease, refers to a group of breathing disorders, in which chronic changes in breathing patterns can produce dyspnea and respiratory and/or non-respiratory symptoms [1]. Hyperventilation syndrome (HVS) is the most common form of DB. In this condition, the patient’s breathing rate is higher than the metabolic requirements of the body [2,3], and alveolar ventilation is excessive, leading to a decrease in arterial carbon dioxide tension (hypocapnia (and an increase in blood pH (respiratory alkalosis) [4]. Hyperventilation usually occurs as a reaction to emotional states, such as anxiety, depression, and stress [5], followed by symptoms including breathlessness, chest pain, and feeling lightheaded [6]. One of the most common tools to diagnose HVS is the Nijmegen questionnaire. This questionnaire is a self-report 16-symptom scale. The frequency of symptoms can be indicated by the following options (score): never (0), rarely (1), sometimes (2), often (3), and very often (4). This questionnaire was developed to screen individuals complaining of shortness of breath with several signs and symptoms of HVS, such as tense feeling, dizziness, fast and deep breathing, feeling of tightness around the mouth, and anxiety. Individuals with a higher score (usually>23 out of 64) on this questionnaire are more prone to HVS. However, there is no gold standard for detecting HVS, and consequently, there is no precise cut-off score for differentiating patients with HVS from individuals without HVS. Moreover, the optimal cut-off score of the Nijmegen questionnaire does not always apply to all populations, and the mean score is dependent on the culture where the questionnaire is being employed [7]. These limitations, especially the absence of a gold standard for the diagnosis of HVS based on the Nijmegen questionnaire, lead to inaccurate estimates of its prevalence. Nevertheless, the prevalence of HVS is estimated at 6-10% in the general population, with a higher prevalence reported in women than men [8-11]. The burden of all DB types, including HVS, is very heavy on patients. In addition, these conditions are associated with significant morbidity, which can be prevented by an accurate diagnosis. Misdiagnosis of HVS, resulting from incorrect cut-off score determination, may lead to inappropriate treatment and higher costs for the patients [12]. Generally, patients with HVS use a significant amount of hospital resources and emergency services, which are very costly; therefore, it is important for paramedics to avoid these problems by determining the exact cut-off score of Nijmegen questionnaire [13]. From a statistical perspective, there are different ways to evaluate the accuracy of a method to diagnose the disease status (HVS patients versus normal individuals) in the absence of a gold standard. One of the recent methods is the Bayesian estimation of disease status, which can provide accurate measures in the absence of a gold standard [14,15]. Therefore, in the current study, we aimed to evaluate the accuracy of the Nijmegen questionnaire in detecting HVS and to provide the best cut-off score for differentiating patients with HVS from normal individuals in Iran using a Bayesian model.

## Materials and Methods

This cross-sectional study was conducted on 490 students of a rehabilitation center affiliated to Shahid Beheshti University of Medical Sciences, Tehran, Iran, using the Nijmegen questionnaire from January to August 2018. The most important eligibility criterion was the absence of asthma; therefore, patients with asthma were excluded from the study. Census sampling was carried out, and a larger sample size than similar studies was selected. All students were asked to participate in an HVS case study. The Iranian version of the Nijmegen questionnaire was used with an internal consistency of 0.7 and acceptable validity, which was not clearly reported [16]. This questionnaire consists of 16 items rated on a five-point Likert scale, with scores in the range of 0-4: never (0), rarely (1), sometimes (2), often (3), and very often (4). This questionnaire evaluates three factors, with seven, four, and five questions, respectively. The total score is within the range of 0-64. If a non-asthma individual obtains a score above 23 (out of 64), he/she will be diagnosed with HVS. A total of 215 non-asthma students completed and returned the questionnaires (response rate=43%). All of the questionnaire responses were reviewed by medical evaluators, and a score was given to each subject. Also, the patients' demographic variables, such as sex, body mass index (BMI), and history of respiratory disease, were recorded. In this study, a simple Bayesian model was developed in order to estimate the test properties, such as sensitivity and specificity, for detecting HVS in the absence of gold standard. In this Bayesian model, a single continuous test was used, in which the non-informative prior distribution of prevalence/sensitivity/specificity were determined by beta distribution (1, 1). The test results are presented as ( *x*_1_,..., *x*_n_), where *x*_i_=1 and *x*_i_=0 denote individuals with and without the disease, respectively. The continuous score of each patient and each normal subject had distributions of N‌(μ^+^,σ^2+^) and N(μ^-^,σ^2-^), respectively. The parameters of this normal distribution were defined as follows:

μ+^+^~‌ N(μ_0_^+^,σ_μ_^2+^) and σ^+^ ~ U(σ_1_^+^,σ_μ_^+^)

μ^-^ ~ N(μ_0_^-^,σ_μ_^2+^) σ^+^ ~ U(σ_1_^+^,σ_μ_^-^)

Where μ_0_^+^(σ_μ_^2+^) and μ_0_^-^(σ_μ_^2-^) are the prior mean (variance) of continuous test results for non-HVS and HVS subjects. The precision of sensitivity and specificity measurements can be determined based on Mont Carlo error and standard deviation (SD) estimations; the latter was used in the current study [15,17]. Furthermore, a semi-parametric approach was applied to estimate the receiver operating characteristic (ROC) curve for continuous test results. The accuracy of each cut-off score for detecting HVS was also determined [18]. This study was approved by the Ethics Committee of Shahid Beheshti University of Medical Sciences (IR.SBMU.REC.1397.507). All analyzes were performed in R version 3.6.2 and OpenBUGS version 3.2.3 and p-value less than 0.05 was considered as significant.

## Results

**Figure 1:**
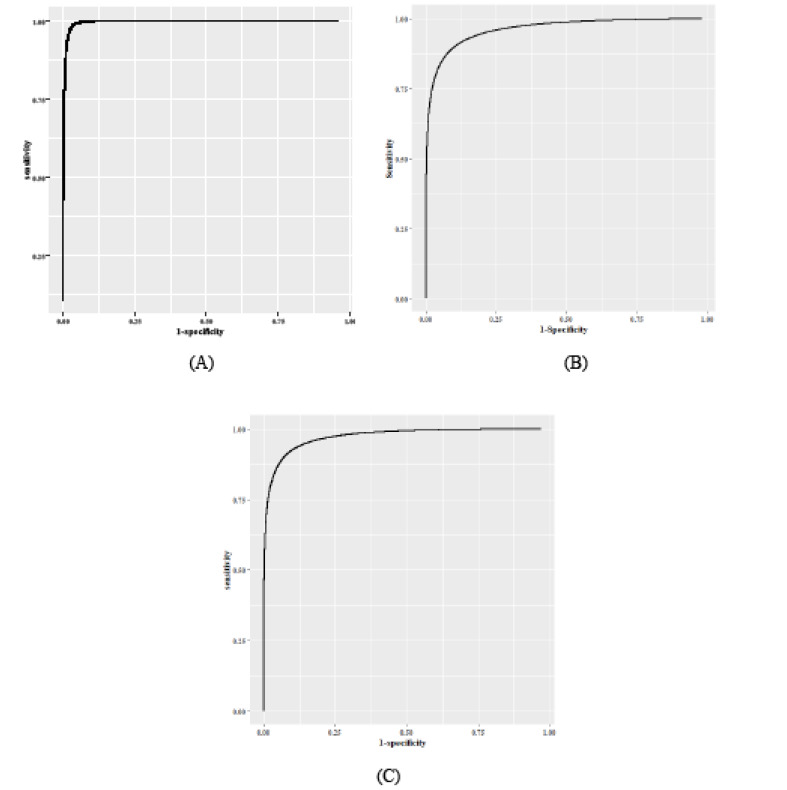
The intersection of sensitivity and specificity for diagnosing HVS in (A) male, (B) female and (C) Total cases

**Figure 2:**
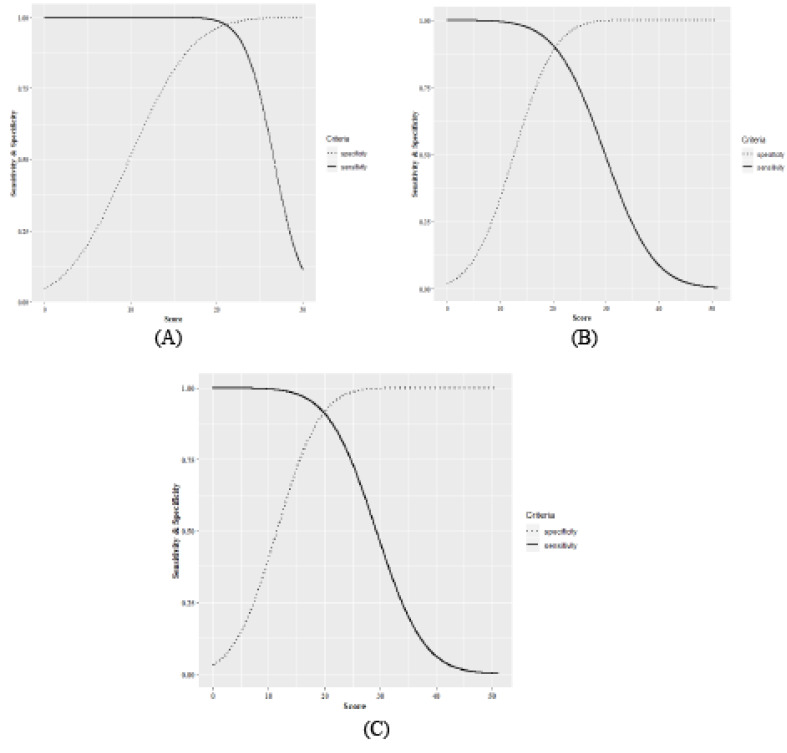
The receiver operating characteristic (ROC) curve of the Nijmegen questionnaire’s score in (A) male, (B) female and (C) Total cases

 A total of 215 students completed the Nijmegen questionnaire from January to August. The HVS and non-HVS groups included 22 (89.8%) and 193 (89.8%) students with the mean±SD scores of 29.32±6.59 and 11.42±6.14, respectively. Among 129 (60.0%) female students, 18 (14.0%) HVS patients were diagnosed, with the mean±SD score of 29.89±7.10. The mean score of 111 (86.0%) non-HVS female patients was 12.59±6.14. Among 86 (40.0%) male cases, 4 (4.7%) and 82 (95.3%) were HVS and non-HVS subjects, respectively. In addition, the mean questionnaire score was 26.75±2.75 in the HVS group and 9.84±5.81 in the non-HVS group. BMI as another characteristic, was evaluated in male/female and HVS/non-HVS subjects, with the mean±SD scores of 23.71±3.47/21.95±305 and 21.86±2.26/22.75±3.43, respectively (Table-1). In the next step, the Bayesian model was applied to the total score of the Nijmegen questionnaire in order to determine the best cut-off score for the diagnosis of HVS, based on sensitivity and specificity criteria. The results showed that the area under the ROC curve (AUC) was 0.93 (male: 0.95; female: 94) for all of the cut-off scores, which indicates the desirable performance of the diagnostic test. In addition, the best cut-off score, based on the intersection of sensitivity and specificity lines, ranged from a total score of 19 to 21 (Figure-1 and Figure-2). According to the Youden index, the optimal cut-off score for HVS prognosis was 20 with sensitivity of 0.91 (male: 0.99; female: 0.91) and specificity of 0.92 (male: 0.96; female: 0.89). With the exception of sensitivity in men, SD of sensitivity and specificity for all criteria was below 0.1, which indicates the high precision of estimations. The highest sensitivity and specificity for differentiating HVS from non-HVS subjects were observed for the cut-off scores of 17 and 23, respectively. Furthermore, there was no significant difference in terms of sensitivity and specificity between the cut-off scores of 19-22 (Table-2).

**Table 1:** The Summarizing of Questionnaire Characteristics by Sex and Disease Status

**Characteristics**	**Category**	**Disease Status**		
		**Non-HVS**	**HVS**	**Total**
		**Mean ± SD/ N (%)**	**Mean ± SD/ N (%)**	**Mean ± SD/ N (%)**
**Sex**	Male	82 (95.3%)	4 (4.7%)	86 (100.0%)
	Female	111 (86.0%)	18 (14.0%)	129 (100.0%)
	Total	193 (89.8%)	22 (10.2%)	215 (100.0%)
**BMI**	Male	23.74 ± 3.55	23.02 ± 1.31	23.71 ± 3.47
	Female	22.01 ± 3.15	21.60 ± 2.37	21.95 ± 3.05
	Total	22.75 ± 3.43	21.86 ± 2.26	22.66 ± 3.33
**Total Score**	Male	9.84 ± 5.81	26.75 ± 2.75	10.63 ± 6.73
	Female	12.59 ± 6.14	29.89 ± 7.10	15.00 ± 8.68
	Total	11.42 ± 6.14	29.32 ± 6.59	13.25 ± 8.23

**Table 2:** The Sensitivity and Specificity of Differentiating HVS to Non-HVS for Different Cut-off Points

**Group**	**Criteria**	**SD ** **of estimates**	**Cut-offs**							**AUC**
			**17**	**18**	**19**	**20**	**21**	**22**	**23**	
Mal **e**	Sensitivity	0.16	0.99	0.99	0.99	0.99	0.97	0.95	0.91	0.95
	Specificity	0.01	0.89	0.92	0.94	0.96	0.97	0.98	0.99	
Female	Sensitivity	0.06	0.96	0.94	0.93	0.91	0.88	0.85	0.82	0.94
	Specificity	0.01	0.76	0.81	0.85	0.89	0.91	0.94	0.95	
Total	Sensitivity	0.05	0.96	0.95	0.93	0.91	0.88	0.85	0.82	0.93
	Specificity	0.01	0.82	0.86	0.89	0.92	0.94	0.96	0.97	

## Discussion

The findings of this study showed that according to AUC (0.93), differentiation of HVS from non-HVS was desirable in all students, based on the total score of Nijmegen questionnaire in the absence of a gold standard. In addition, the optimal cut-off score for the detection of HVS was 20. The AUC of Nijmegen questionnaire for diagnosis of HVS was higher in male individuals, compared to females; however, both genders had a similar optimal cut-off score to the total population of students. Our findings showed that the mean Nijmegen score of females was higher than that of males in both HVS and non-HVS groups. Our findings showed that HVS patients were mostly women, with mildly lower BMI. These findings are consistent with studies, which reported a higher prevalence of HVS in women [2,19,20]. In this regard, Cowley and Roy-Byrne showed that the prevalence of HVS was the highest in patients with psychological pathologies. In addition, mental disorders had a higher prevalence among women than men [21,22]. Therefore, one of the reasons for the higher prevalence of HVS in women may be the higher prevalence of mental disorders in this group. In the current study, there was no gold standard for HVS; consequently, the mean scores of the Nijmegen questionnaire were not precise in HVS and non-HVS individuals. However, the total mean score was lower than that reported in asthmatic patients in similar studies [9,16]. This may be due to poor asthma control, which leads to an increase in the score of the Nijmegen questionnaire [8,9,23]. Consequently, a single cut-off score cannot be considered for all HVS patients, and the patient’s initial information should be taken into consideration in order to determine the best cut-off score, as used in our model. In almost all studies in this area, differentiation of patients with hyperventilation from others is accomplished using the Nijmegen questionnaire, and AUC is usually more than 0.9 [9,24]. The findings of the present study were in agreement with the aforementioned studies, and the estimated AUC exceeded 0.90 in males and females, indicating the high capacity of the Nijmegen questionnaire in differentiating patients with HVS from others in the absence of a gold standard. In this study, it was found that the Nijmegen questionnaire with a cut-off score of ≥20 could diagnose patients with HVS, using a Bayesian model in the absence of a gold standard, with the estimated sensitivity and specificity of 91% and 92%, respectively. Our findings are not in agreement with studies conducted by Grammatopoulou el al., in which the cut-off score of ≥18 differentiated patients with HVS from normal individuals, with 92.73% sensitivity and 91.59% specificity [9]. Another study by Dixhoorn and Folgering showed an optimal cut-off score of ≥19, with 90% correct classification of HVS [7]. Moreover, in some studies, various cut-off scores, such as 22 and 23, have been used [8,10,25,26,27]. This discrepancy could be due to the absence of a gold standard for the Nijmegen questionnaire and inclusion of asthmatic patients in the study, which can increase the Nijmegen questionnaire score. However, our findings showed that the accuracy of cut-off scores ≥20 and 19 were very close. There were some potential limitations in the current study, such as non-random selection of samples and non-inclusion of asthmatic patients in the study, which can prevent generalization of our results to other populations. On the other hand, this is the first study measuring AUC to provide the best cut-off score in the absence of a gold standard, using the Nijmegen questionnaire.

## Conclusion

 Based on the findings, accurate differentiation of HVS from non-HVS individuals can be performed based on the estimated cut-off score of ≥20 on the Nijmegen questionnaire, using a Bayesian model.

## Conflict of Interest

 The authors have no conﬂicts of interest in writing this article.

## References

[1] Barker N, Everard ML (2015). Getting to grips with 'dysfunctional breathing'. Paediatr Respir Rev.

[2] Gardner WN (1996). The pathophysiology of hyperventilation disorders. Chest.

[3] Courtney R, Greenwood KM, Cohen M (2011). Relationships between measures of dysfunctional breathing in a population with concerns about their breathing. J Bodyw Mov Ther.

[4] Robson A (2017). Dyspnoea, hyperventilation and functional cough: a guide to which tests help sort them out. Breathe (Sheff).

[5] Connett GJ, Connett LA, Thomas M (2019). Determining the reasons for poorly controlled asthma in an adolescent. BMJ.

[6] Hyperventilation: Symptoms C, Treatment, Emergencies. WebMD, 2019. (Accessed October 21, 2019, at https://www.webmd.com/lung/lung-hyperventilation-what-to-do#1-3).

[7] van Dixhoorn J, Folgering H (2015). The Nijmegen Questionnaire and dysfunctional breathing. ERJ Open Res.

[8] Thomas M, McKinley RK, Freeman E, Foy C (2001). Prevalence of dysfunctional breathing in patients treated for asthma in primary care: cross sectional survey. BMJ.

[9] Grammatopoulou EP, Skordilis EK, Georgoudis G, Haniotou A, Evangelodimou A (2014). Hyperventilation in asthma: a validation study of the Nijmegen Questionnaire--NQ. J Asthma.

[10] Thomas M, McKinley RK, Freeman E, Foy C, Price D (2005). The prevalence of dysfunctional breathing in adults in the community with and without asthma. Prim Care Respir J.

[11] Vidotto LS, Carvalho CRFd, Harvey A, Jones M (2019). Dysfunctional breathing: what do we know?. J Bras Pneumol.

[12] Depiazzi J, Everard ML (2016). Dysfunctional breathing and reaching one's physiological limit as causes of exercise-induced dyspnoea. Breathe (Sheff).

[13] Wilson C (2018). Hyperventilation syndrome: diagnosis and reassurance. JPP.

[14] Ling DI, Pai M, Schiller I, Dendukuri N (2014). A Bayesian framework for estimating the incremental value of a diagnostic test in the absence of a gold standard. BMC Med Res Methodol.

[15] Joseph L, Gyorkos TW, Coupal L (1995). Bayesian estimation of disease prevalence and the parameters of diagnostic tests in the absence of a gold standard. Am J Epidemiol.

[16] Ravanbakhsh M, Nargesi M, Raji H, Haddadzadeh Shoushtari M (2015). Reliability and Validity of the Iranian Version of Nijmegen Questionnaire in Iranians with Asthma. Tanaffos.

[17] Ladouceur M, Rahme E, Belisle P, Scott AN, Schwartzman K, Joseph L (2011). Modeling continuous diagnostic test data using approximate Dirichlet process distributions. Stat Med.

[18] Cai T, Moskowitz CS (2004). Semi-parametric estimation of the binormal ROC curve for a continuous diagnostic test. Biostatistics.

[19] Brat K, Stastna N, Merta Z, Olson LJ, Johnson BD, Cundrle I, Jr Jr (2019). Cardiopulmonary exercise testing for identification of patients with hyperventilation syndrome. PloS one.

[20] Pfortmueller CA, Pauchard-Neuwerth SE, Leichtle AB, Fiedler GM, Exadaktylos AK, Lindner G (2015). Primary Hyperventilation in the Emergency Department: A First Overview. PLoS One.

[21] Steel Z, Marnane C, Iranpour C, Chey T, Jackson JW (2014). The global prevalence of common mental disorders: a systematic review and meta-analysis 1980-2013. Int J Epidemiol.

[22] Charlson F, van Ommeren M, Flaxman A, Cornett J, Whiteford H, Saxena S (2019). New WHO prevalence estimates of mental disorders in conflict settings: a systematic review and meta-analysis. The Lancet.

[23] de Groot EP, Duiverman EJ, Brand PL (2013). Dysfunctional breathing in children with asthma: a rare but relevant comorbidity. Eur Respir J.

[24] Talaat HS, Moaty AS, Elsayed MA (2019). Arabization of Nijmegen questionnaire and study of the prevalence of hyperventilation in dizzy patients. Hearing Balance Commun.

[25] Stanton AE, Vaughn P, Carter R, Bucknall CE (2008). An observational investigation of dysfunctional breathing and breathing control therapy in a problem asthma clinic. J Asthma.

[26] Demeter SL, Cordasco EM (1986). Hyperventilation syndrome and asthma. Am J Med.

[27] Agache I, Ciobanu C, Paul G, Rogozea L (2012). Dysfunctional breathing phenotype in adults with asthma - incidence and risk factors. Clin Transl Allergy.

